# Electrophysiological Correlates of Cue-Related Processing in a Gambling Task: Early Outcome Evaluation or Outcome Expectation?

**DOI:** 10.3389/fpsyg.2017.00978

**Published:** 2017-06-13

**Authors:** Xiaoyi Zhang, Xiaohong Lin, Shiho Takagi, Liyang Sai

**Affiliations:** ^1^School of Education Science, Huangshan UniversityHuangshan, China; ^2^Bioimaging Core, Faculty of Health Sciences, University of MacauMacau, China; ^3^School of Psychology and Cognitive Science, East China Normal UniversityShanghai, China; ^4^Institutes of Psychological Sciences, Hangzhou Normal UniversityHangzhou, China; ^5^Zhejiang Key Laboratory for Research in Assessment of Cognitive ImpairmentsHangzhou, China; ^6^Center for Cognition and Brain Disorders, Hangzhou Normal UniversityHangzhou, China

**Keywords:** feedback-related negativity, event-related potentials, outcomes expectation, outcome evaluation, predictive cues

## Abstract

Several recent studies have suggested that cues that predict outcomes elicit a feedback-related-like negativity (FRN-like negativity) reflecting initial appraisals of whether desired outcomes are probable. Some other studies, however, have found that the cues that predict outcomes elicited event-related potentials (ERPs) that reflect the expectation to outcomes (e.g., outcome expectations). Given these mixed findings, this study aimed to examine whether the brain activity elicited by predictive cues in a gambling task reflected the initial evaluations of the outcomes, the outcome expectations, or both. We used a gambling task in which the participants were told to guess which of two doors hid a reward. At the beginning of each trial, a cue was presented to inform the participants of how many doors hid a reward. We found that these predictive cues elicited a FRN-like negativity at the frontal sites within around 200–300 ms. However, this negativity did not significantly differ between the cues that fully predicted gains and the cues that fully predicted losses. Furthermore, predictive cues elicited an expectation-related slow wave, and cues that predicted gains with a 50% probability elicited a larger expectation-related slow wave than cues that fully predicted gains or losses. Our results suggest that cues predicting outcomes reflect outcome expectations rather than initial evaluations of the forthcoming outcomes.

## Introduction

To behave adaptively, individuals must evaluate the outcomes of their decisions, and must use their evaluations to guide reward-seeking behavior. In the last decade, a great number of ERP studies have investigated the neural mechanisms of outcome evaluation (For a review, see Walsh and Anderson, 2012). These studies have consistently identified a feedback-related negativity (FRN) that appears 200–300 ms after feedback, and this FRN is larger for unfavorable outcomes than for favorable outcomes (Gehring and Willoughby, 2002; Hajcak et al., 2005). Reinforcement learning-error-related negativity (RL-ERN) theory has proposed that the FRN reflects the reward prediction errors made during reinforcement learning (Holroyd and Coles, 2002). According to RL-ERN theory, the basal ganglia monitors and predicts outcomes, and when outcomes are better or worse than expected, this reward prediction error (the discrepancy between an outcome and a prior prediction) induces a phasic increase or decrease in the activity of the midbrain dopamine neurons. The dopamine signals are then conveyed to the anterior cingulate cortex (ACC), where they are used as reinforcement learning signals to adjust behavior. RL-ERN theory proposes that the FRN reflects the effect of this phasic dopamine signal on the ACC.

As the dopamine response transfers back in time from outcomes to the earliest events that predict those outcomes (Schultz et al., 1997; Schultz, 2007), ERN-reinforcement learning theory holds that events predicting outcomes would elicit a frontal negativity. Recent evidence has supported this prediction (Dunning and Hajcak, 2007; Baker and Holroyd, 2009; Holroyd et al., 2011; Liao et al., 2011). For example, Dunning and Hajcak (2007) found that cues that fully predicted further losses elicited a larger negativity than cues that fully predicted further wins. Holroyd et al. (2011) reported that cues that predicted upcoming rewards with an 80% probability elicited a larger reward positivity than cues that predicted no reward with an 80% probability^1^. These studies suggested that the FRN-like component, elicited by predictive cues, reflects an initial appraisal of whether the desired outcomes are probable (*early outcome evaluation*).

On the other side, cues can inform individuals about the probability of obtaining rewards, and individuals are likely to use such information to shape their levels of expectation concerning forthcoming outcomes (Knutson et al., 2005). In this regard, we would expect that our brain should encode a reward expectation (an anticipation of reward) during the cue period. In fact, this cue-related reward expectation has been extensively studied by using functional magnetic resonance imaging (fMRI) (Knutson et al., 2001, 2008), and more recently by using ERPs (Broyd et al., 2012; Pfabigan et al., 2014; Novak and Foti, 2015). Typically, such studies have used the monetary incentive delay (MID) task. In this paradigm, the participants are asked to respond as quickly as possible by pressing a button related to a target. Before the target is presented, a cue is given to indicate whether responding to the target relates to an attempt to win money, to avoid losing money, or to break even. Unlike the ERP studies that have found cues predicting outcomes elicited a FRN-like negativity, these studies have found that cues predicting outcomes elicited a larger P300, or a larger slow wave (e.g., a contingent negative variation, also called a CNV) than neutral cues. These studies have also suggested that the brain activity (e.g., P300/CNV) that is elicited by predictive cues reflects a *reward expectation* (an expectation of a reward/outcome).

Given these mixed findings about the neural correlates associated with cue-related processing (early outcome evaluation vs. reward expectation), this study examined whether brain activity during the cue period reflects an initial evaluation of the forthcoming outcome (i.e., an evaluation of whether the desired outcomes are probable), or a reward/outcome expectation, or both. It should be noted that studies that support the idea that the brain activity reflects an initial evaluation of a forthcoming outcome have typically found that an FRN effect occurs around 200–300 ms after the cue presentation. However, studies which have supported the idea that the brain codes reward expectation have usually found a P300 or CNV after 300 ms. These differing results may suggest that when a cue is presented, the brain does an initial evaluation of forthcoming outcomes first, and then uses cue information to form a reward expectation (also see, Osinsky et al., 2016). For example, when cues are presented, individuals may first make an initial appraisal about whether the desired outcomes are probable (early outcome evaluation), and then use the information provided by the cues to shape their expectation regarding any upcoming reward (reward expectation).

To test this hypothesis, we used a gambling task in which the participants were told to guess which of two doors hid a reward (Dunning and Hajcak, 2007). At the beginning of each trial, a cue (which could be a 0, 1, or 2) was presented, to inform the participants regarding the number of doors that hid a reward (This stage in the task was called the cue period). Cues 0 and 2 predicted losses or gains, respectively, with 100% accuracy. The cue 1 predicted gains with 50% accuracy. Each participant was then asked to guess where the reward was by selecting one of two doors. After the participant made a choice, the outcome was presented (the feedback period).

We expected that the cues that predicted outcomes would elicit not only an FRN-like negativity, reflecting the initial appraisal of whether the desired outcomes was probable (e.g., Holroyd et al., 2011), but also an ERP component (P300 or CNV), reflecting an expectation concerning the upcoming outcome (e.g., Broyd et al., 2012; Pfabigan et al., 2014; Silvetti et al., 2014; Novak and Foti, 2015; Osinsky et al., 2016).

## Materials and methods

### Participants

Twenty-three undergraduates from Zhejiang Normal University in China (11 females, mean age = 20.30 years, SD = 2.06 years) were paid for participation. All of the participants had normal or corrected-to-normal vision, and were right-handed. Written informed consent was obtained from each participant included in the study. The study was approved by the Ethics Committee of Zhejiang Normal University.

### Gambling task

We used a gambling task similar to the task used by Foti and Hajcak (2012). During each trial, an image was presented on the computer screen, showing two doors side-by-side. The participants were then asked to choose one of the doors by pressing either the left or right button. After the participants chose a door, a feedback screen appeared that indicated whether the participants had won ¥4 (about 0.7 dollars) or lost ¥2 (about 0.35 dollars). Before seeing the image with the two doors, the participants saw one of three cues: 0, 1, or 2. These cues indicated the number of doors that contained a reward. The cues 0, 1, or 2 indicated that the probabilities of winning a reward in the upcoming trial were 0, 50, or 100%, respectively.

Each participant sat about 1 m in front of a computer monitor. The stimuli appeared in black font on a white background. Each trial began with a cue (lasting 1,000 ms). After a fixation period (of 500 ms), the image of the two doors was presented. The doors remained on the screen until the participant made a response. When the participant chose a door, another fixation was presented for 3,000 ms, followed by the feedback (2,000 ms) for the choice. After the feedback, another fixation was presented for 1,000 ms. The participants then pressed a button to start the next trial. There were 160 trials in total (40 having a 0 cue, 80 having a 1 cue, and 40 having a 2 cue), which were presented randomly.

### EEG acquisition

The EEG data were recorded by 32 Ag/AgCl electrodes (international 10–20 arrangement) embedded in an elastic cap (Neuroscan Inc., USA). On-line recordings were referenced to the left mastoid, and the data were then re-referenced offline to the mean of the left and right mastoids. The electrode impedances were kept below 5 kΩ. The vertical electro-oculograms (EOGs) were recorded above and below the right eye, and the horizontal EOGs were recorded from electrodes placed at the outer canthi of the left and right eyes. The EEG and the EOG were sampled at 500 Hz.

For offline analyses, continuous EEGs were first filtered with a low-pass filter (30 Hz cut-off, 24 dB/ct) and a high-pass filter (0.1 Hz cut-off, 48 dB/ct). For the cue period, continuous EEGs were segmented into epochs from –200 to 1,000 ms, with a time of 0 ms locked to the cue stimuli. Baseline correction was performed for each trial, using a 200 ms prior-to-stimuli onset. Then, the cue-locked ERPs were separately averaged for the trials of cues 0, 1, and 2. The amplitude of the FRN-like negativity during the cue period was calculated as the mean amplitude of 250–350 ms, after cue presentation, at Fz, FCz, and Cz. The slow negative component was calculated as the mean amplitude of 800–1,000 ms, after cue presentation, at Fz, FCz, and Cz. The frontal-central area was chosen for measuring the slow negative component, because one recent study has suggested that the expectation-related slow negative component during the cue period is generated in the frontal cortex (Silvetti et al., 2014). The feedback-locked ERPs were separately averaged for 0-cue loss, 1-cue gain, 1-cue loss, and 2-cue gain trials. The FRN was calculated by using the difference between the loss and gain trials (loss minus gain), and it was averaged between 280 and 380 ms after the feedback onset at Fz, FCz, and Cz (Bress and Hajcak, 2013). We used the ocular artifact reduction algorithm (ARTCOR procedure) in Scan 4.3 to remove ocular artifacts (also see Groen et al., 2008). Trials with artifacts exceeding ± 100 μV were excluded from averaging.

## Results

### The ERPs in the cue period

#### FRN-like negativity

A three (cue type: 0, 1, and 2) by three (electrode site: Fz, FCz, and Cz) within-subject repeated measure ANOVA was conducted on the amplitude of FRN-like negativity in the cue period. The results showed a significant main effect of cue type, *F*_(1, 22)_ = 3.391, *p* = 0.043, $\eta_{p}^{2}$ = 0.134, observed power = 0.609. Further analysis found that the 0-cue trials elicited a more negative FRN-like negativity (M = 5.36 μV, SD = 0.84) than the 1-cue trials (M = 6.5 μV, SD = 0.82), *p* = 0.035. Also, the 2-cue trials elicited a stronger FRN-like negativity (M = 5.28 μV, SD = 0.76) than the 1-cue trials (M = 6.5 μV, SD = 0.82), *p* = 0.02. However, there was no significant difference in the FRN-like negativity elicited by the 0-cue trials and the 2-cue trials, *F* < 1, *p* > 0.1. Neither the main effects of the electrode sites nor the interaction between the cue types and the electrode sites were significant (*Fs* < 1, *ps* > 0.1).

#### Slow negative wave

**Figure 1:**
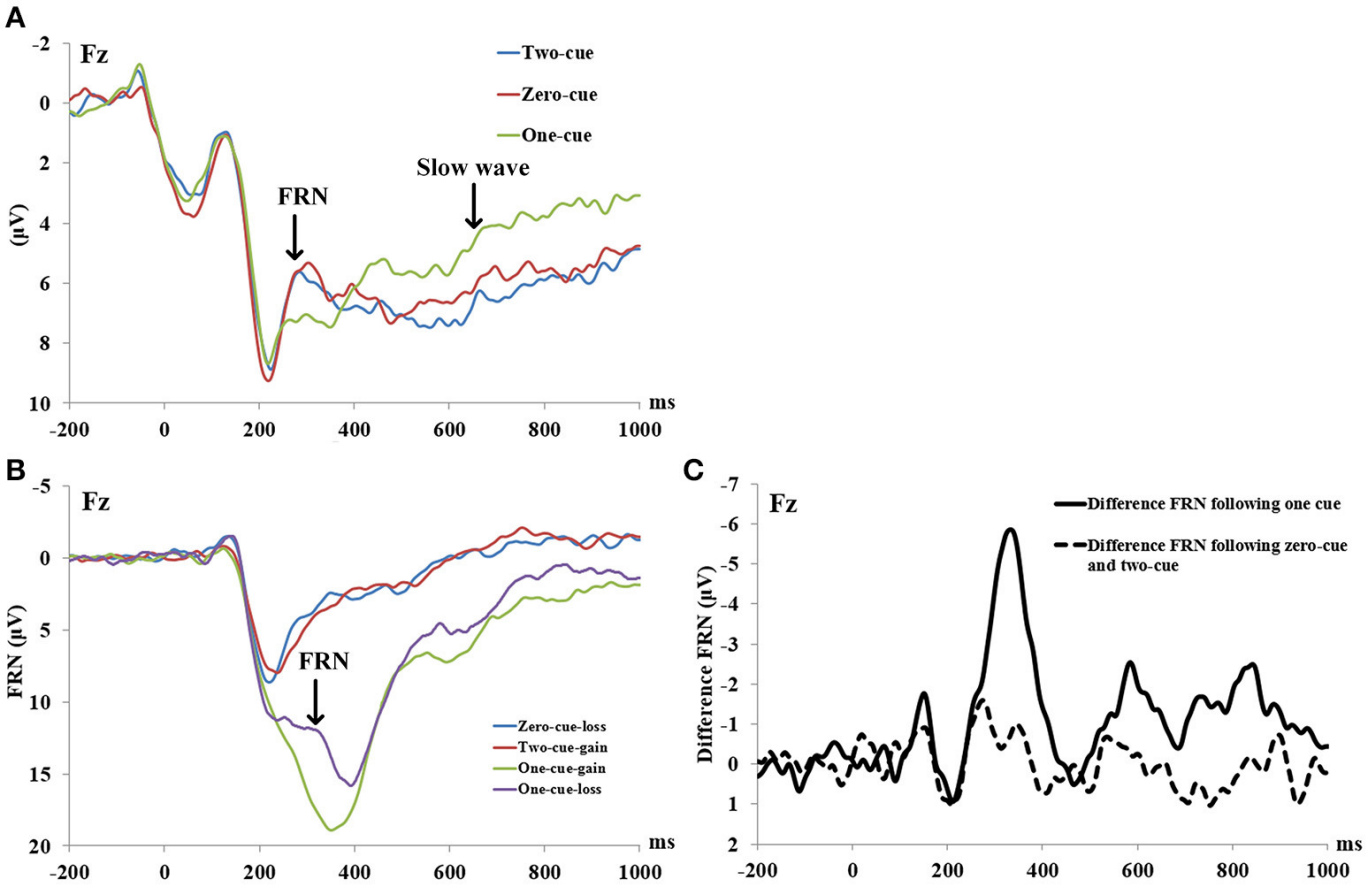
**(A)** ERP waveforms during the cue period at electrodes Fz. Cue onset was at 0 ms. **(B)** ERP waveforms during the outcome evaluation period at electrodes Fz. The feedback onset was at 0 ms. **(C)** Difference waves during the outcome evaluation period at electrodes Fz. The feedback onset was at 0 ms.

**Figure 2:**
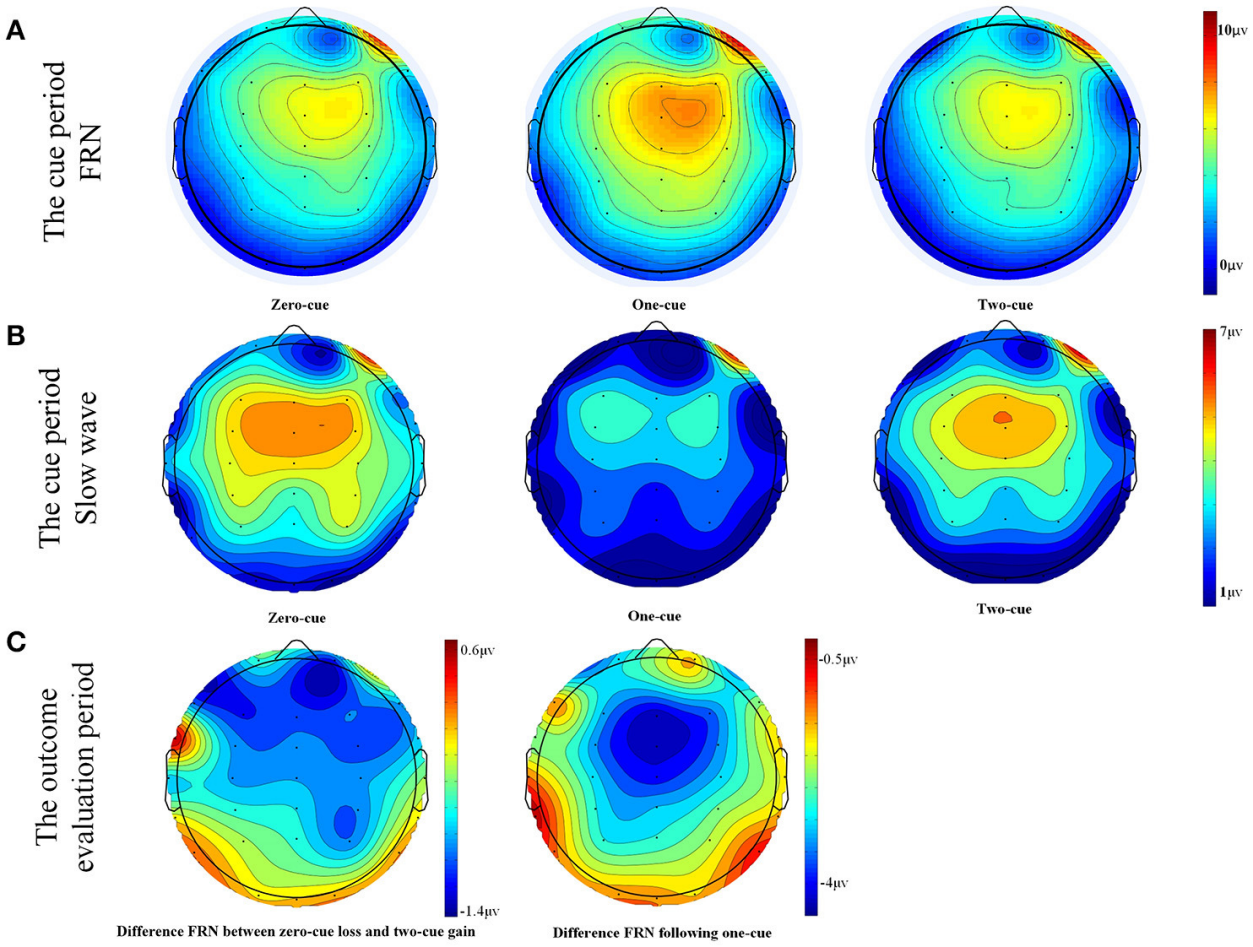
**(A)** Scalp topographies for each condition in the time window of 200–300 ms during the cue period. **(B)** Scalp topographies for each condition in the time window of 800–1000 ms during the cue period. **(C)** Scalp topographies for differences in waves during the time window of 280–380 ms in the outcome evaluation period.

Furthermore, scalp topography showed a frontal-central slow wave being apparent around 800–1,000 ms after the cue presentation. A three (cue type: 0, 1, and 2) by three (electrode site: Fz, FCz, and Cz) within-subject repeated measure ANOVA was conducted on the amplitude of the slow wave after cue presentation. The results showed a significant main effect of cue type, *F*_(1, 22)_ = 8.528, *p* = 0.001, $\eta_{p}^{2}$ = 0.279, observed power = 0.956. The 1-cue trials elicited a more negative component (M = 2.505 μV, SD = 0.94) than the 0-cue trials (M = 4.782 μV, SD = 0.93), or the 2-cue trials (M = 4.804 μV, SD = 1.2) (With the *p*-values being *p* = 0.001 and 0.002, respectively). However, there was no significant difference between the 0-cue trials and the 2-cue trials (*p* > 0.5). Neither the main effect of the electrode site nor the interaction between the cue type and electrode site were significant, *F*_(1, 22)_ = 0.257, *p* = 0.775, $\eta_{p}^{2}$ = 0.012; *F*_(1, 22)_ = 0.194, *p* = 0.941, $\eta_{p}^{2}$ = 0.009. For the grand-averaged ERPs and their scalp distributions that were locked to cues, see Figures 1A, 2A.

### The ERP in the outcome evaluation period

A two (outcomes: the difference in FRN following a 2 cue and a 0 cue vs. the difference in FRN following a 1 cue) by three (electrode site: Fz, FCz, and Cz) within-subject repeated measure ANOVA was conducted on the differences in FRN amplitude. The results revealed a significant main effect of outcomes, *F*_(1, 22)_ = 18.681, *p* < 0.001, $\eta_{p}^{2}$ = 0.46, observed power = 0.96, with a larger difference in FRN following a 1 cue (M = 4.52 μV, SD = 0.66) than a 2 and a 0 cue (M = 0.40 V, SD = 0.66). No other significant main or interaction effect was found (*F*s ≤ 2, *ps* > 0.06). For grand-averaged ERPs and their scalp distributions that were locked to feedback, see Figures 1B,C, 2B,C.

## Discussion

### FRN-like negativity

In this study, we used ERPs in a modified gambling task to investigate whether brain activities that were elicited by predictive cues reflected early evaluations of forthcoming outcomes, outcomes expectations, or both. As was consistent with prior studies and with our prediction, we found a negative component at around 200–300 ms after cue presentation (Dunning and Hajcak, 2007; Holroyd et al., 2011; Novak and Foti, 2015). However, further analysis showed no significant differences for this negativity between cues that fully predicted losses (the 0-cue condition) and cues that fully predicted gains (the 2-cue condition). This result was not consistent with some of the previous studies or with our prediction (Holroyd et al., 2011; Novak and Foti, 2015). For example, Holroyd et al. (2011) found that cues that predicted upcoming rewards with 80% probability elicited a larger reward positivity than cues that predicted no reward with 80% probability. Novak and Foti (2015) found that cues that predicted potential losses elicited a larger negativity than cues that predicted potential gains. However, this finding that there were no significant differences for FRN between cues that predicted losses (the 0-cue condition) and cues that predicted gain (the 2-cue condition) was consistent with two other previous studies (Nieuwenhuis et al., 2007; Osinsky et al., 2016). It should be noted that the cues that predicted outcomes were with a certain probability in some of those studies that have found cues predicting losses elicited a larger negativity than cues predicting gains (e.g., 80% in study by Holroyd et al., 2011). In some other of those studies, participants needed to learn the meanings of the cues. For example, in the study by Osinsky et al. (2014), the participants needed to learn which prosper is fair/unfair during an Ultimatum Game before the faces of prospers became valid predictive cues for the inequity of the pending offers (also see Baker and Holroyd, 2009). However, the cues used in this study fully predicted gains or losses, and the participants were told the meaning of each cue before the experiment, which made the cues perfectly predictive of the outcomes. Therefore, cues predicting losses and those predicting gains did not significantly differ in this study (also see, Dunning and Hajcak, 2007). Further studies should test this hypothesis by conducting an experiment without telling the participants the meanings of each cue beforehand.

### Slow negative wave

As was consistent with our prediction, we found that a slow negative wave arose after cue presentation. This result was consistent with the results from several previous studies that have reported CNV arising after the onset of cues (Goldstein et al., 2006; Silvetti et al., 2014; Novak and Foti, 2015; Osinsky et al., 2016). Furthermore, the slow negative wave was larger for cues that predicted a loss with 50% probability than for cues that predicted either a loss or a gain with 100% probability. This result was compatible with another finding by Fiorillo et al. (2003). Using single-unit recording, these researchers found that sustained, anticipatory activation of dopamine neurons varied with reward probability. Their results showed larger responses for uncertain conditions (50% reward probability), and that responses grew smaller when the probability of receiving a reward became higher or lower. Our results were also in line with those of Foti and Hajcak (2012), who found that another expectation-related ERP component, namely the stimulus preceding negativity (SPN), was larger for uncertain cues than for certain cues. These results suggested that the cue-linked CNV effect found in our study may reflect uncertainty, and preparatory activation of the performance monitoring functions during reward expectation (also see Osinsky et al., 2016).

### FRN during outcome evaluation

For the outcome evaluation period, we found that cues indicating predictable losses (losses after a 0 cue) did not evoke a significantly larger FRN than cues indicating predictable gains (gains after a 2 cue). However, we found that losses after a 1 cue elicited a larger FRN than gains after a 1 cue. RL-ERN theory holds that FRN reflects the reward prediction error, and its amplitude depends on the difference between the expected and the actual outcomes (Hajcak et al., 2005, 2007). In this study, as both the 0 cue and the 2 cue predicted the actual outcomes with 100% accuracy, there was no difference between the expected and the actual outcomes. Therefore, these cues evoked a small FRN. However, in the 1-cue condition, the participants were asked to try to guess which door of the two doors hid a prize to win the money, after the participants made their choices, they should expect the door they chose hid a prize, and when the actual outcomes were losses, as the large prediction error in these cases elicited larger FRN. These results were in line with those of many previous studies (e.g., Dunning and Hajcak, 2007; Bress and Hajcak, 2013). Taken altogether, these findings suggested that the FRN reflects the reward predicted error.

In summary, this study has examined whether brain activity during the cue period in a gambling task reflects the early outcome evaluation, reward expectations, or both. We found that cues elicited a FRN-like negativity at the frontal site within around 200–300 ms. However, this negativity was not sensitive to valence, but instead was sensitive to reward probability, with the negativity elicited by cues that fully predicted gains or losses being larger than cues that predicted gains with 50% probability. In addition, all cues elicited an expectation-related slow negative wave, with the cues that predicted gains with 50% probability larger than the cues that fully predicted certain gains or certain losses. These findings suggested that the brain activity associated with the cues that predicted outcomes reflected uncertainty, and preparatory activation of the performance monitoring functions associated with reward expectations, rather than initial evaluations of the forthcoming outcomes (early outcome evaluation).

## Ethical approval

All procedures performed in studies involving human participants were in accordance with the ethical standards of the institutional and/or national research committee and with the 1964 Helsinki declaration and its later amendments or comparable ethical standards.

## Author contributions

XZ: Had the idea, did the experiment, and wrote the paper. ST: Did the experiment and wrote the paper. XL: Did the experiment and analyzed the data. LS: Had the idea and wrote the paper.

### Conflict of interest statement

The authors declare that the research was conducted in the absence of any commercial or financial relationships that could be construed as a potential conflict of interest.

## Footnotes

^1^Holroyd et al. (2011) reported a reward positivity elicited by cues that predicted outcomes. This is because they subtracted gain from no gain to measure the FRN. However, studies which reported negativity elicited by cues that predicted outcomes typically subtracted no gain from gain to measure the FRN.
